# Caracterización clínica y epidemiológica de fiebre chikungunya en México

**DOI:** 10.26633/RPSP.2017.58

**Published:** 2017-07-01

**Authors:** Carolina Garay-Morán, Juan Francisco Román-Pedroza, Irma López-Martínez, José Cruz Rodríguez-Martínez, Cuitláhuac Ruiz-Matus, Pablo Kuri-Morales, José Alberto Díaz-Quiñonez

**Affiliations:** 1 Dirección General de Epidemiología Dirección General de Epidemiología México D.F. México Dirección General de Epidemiología, México D.F., México; 2 Instituto de Diagnóstico y Referencia Epidemiológicos “Dr. Manuel Martínez Báez” México D.F. México Instituto de Diagnóstico y Referencia Epidemiológicos “Dr. Manuel Martínez Báez”, México D.F., México.; 3 Subsecretaría de Prevención y Promoción de la Salud Subsecretaría de Prevención y Promoción de la Salud México D.F México Subsecretaría de Prevención y Promoción de la Salud, México D.F., México

**Keywords:** Virus chikungunya, epidemiología, México, Chikungunya virus, epidemiology, Mexico

## Abstract

El 6 de diciembre de 2013, la Organización Panamericana de la Salud (OPS) y la Organización Mundial de la Salud (OMS) notificaron la confirmación de los dos primeros casos de transmisión autóctona en la Región de las Américas de fiebre chikungunya (CHIK) en la isla de Saint Martin (Antillas Neerlandesas). Para el período 2013-2014, el total de casos confirmados fue de 25 627 distribuidos en 43 países, donde México reportó 155 casos en cinco estados. La información de los casos de CHIK en México se obtuvo de la base de datos de la Dirección General de Epidemiología, dependiente de la Secretaría de Salud de México. La distribución por sexo de los casos autóctonos confirmados de CHIK para el año 2015 indica 64% para el sexo femenino (5 583) y 36% para el sexo masculino (3 085). Los síntomas más frecuentes fueron: fiebre en 98% de los casos (8 564), seguido por cefalea con 91,6% (7 941), mialgias en 89,9% (7 792), artralgias leves en 73,5% (6 367), poliartralgias graves en 72,6% (6 295) y exantema en 58% (5 032). La presentación clínica de los casos autóctonos de CHIK en México ha mostrado algunas características clínicas diferentes de las que se han observado en los brotes de los países africanos, asiáticos y otras regiones de América, como por ejemplo un mayor porcentaje de casos con cefalea y mialgias y un menor porcentaje de casos con artralgias.

## Abstract (en)

On 6 December 2013, the Pan American Health Organization (PAHO) and the World Health Organization (WHO) reported confirmation of the first two cases of indigenous transmission of chikungunya fever (CHIK) in the Region of the Americas on the island of Sint Maarten (Netherlands Antilles). For the period 2013-2014, a total of 25 reporting 155 cases in five states. Information on cases of CHIK in Mexico was obtained from the database of the General Directorate of Epidemiology (Ministry of Health of Mexico). The distribution of confirmed autochthonous cases of CHIK for 2015, by sex, was 64% female (5 583) and 36% male (3 085). The most frequent symptoms were fever in 98% of cases (8 564), followed by headache in 91.6% (7 941), myalgia in 89.9% (7 792), mild arthralgias in 73.5% (6 367), severe polyarthralgia in 72.6% (6 295), and exanthema in 58% (5 032). The clinical presentation of autochthonous cases of CHIK in Mexico has shown several clinical manifestations different from those seen in outbreaks in African and Asian countries and other regions in the Americas; for example, a greater percentage of cases with headache and myalgia and a smaller percentage of cases with arthralgia.

La fiebre chikungunya (CHIK) es una enfermedad emergente, causada por un virus de ácido ribonucleico (ARN) de 60 a 70 nm de diámetro perteneciente al género Alphavirus, del grupo A de arbovirus, de la familia Togaviridae (1). El virus chikungunya (CHIKV) se aisló y describió por primera vez en el ser humano durante una epidemia en Tanzania entre 1952 y 1953; luego se han reportado brotes en la India y países de África. Desde el año 2010 se la llegó a considerar como una enfermedad emergente con potencial de epidemia (2). El 6 de diciembre de 2013, la Organización Panamericana de la Salud (OPS) y la Organización Mundial de la Salud (OMS) notificaron la confirmación de los dos primeros casos de transmisión autóctona de CHIK en América en la Isla de Saint Martin (Antillas Neerlandesas) (3). Entre 2013 y 2014, el total de casos autóctonos confirmados fue de 25 627 distribuidos en 43 países, donde México reportó 155 casos en cinco estados del país (4, 5). Para unificar criterios para la detección, notificación y seguimiento de los casos de CHIK, se elaboraron definiciones operacionales de caso aprobadas por el Comité Nacional de Vigilancia Epidemiológica de México (CoNaVE), las cuales fueron publicadas para el libre acceso en el sistema de salud de México. Todos los casos reportados han sido confirmados por métodos de laboratorio por la Red Nacional de Laboratorios de Salud Pública con la referencia del Instituto de Diagnóstico y Referencia Epidemiológicos “Dr. Manuel Martínez Báez” (InDRE) de México (6). La CHIK es una enfermedad emergente en México, lo que indica que no existe un seguimiento sobre las características epidemiológicas de loscasos de fiebre chikungunya. En este estudio se realiza una caracterización epidemiológica con la información disponible.

## MATERIALES Y MÉTODOS

### Recolección de datos

La información de los casos de CHIK en México se obtuvo de la base de datos de la Dirección General de Epidemiología de México a través del Laboratorio de Arbovirus y Virus Hemorrágicos del InDRE, correspondientes a los casos confirmados por laboratorio para CHIKV con corte en la semana epidemiológica 40. Se realizó una depuración de la base y se dejaron solo las variables que se necesitan para el presente estudio, calculando frecuencias y porcentajes aquí reportados.

### Definiciones operacionales

Se consideró como caso sospechoso de CHIK a toda persona que presente cuadro febril agudo más la presencia de poliartralgias graves (incapacitantes) o artritis de comienzo agudo y que se identifique alguna asociación epidemiológica:

**CUADRO 1.** Casos autóctonos confirmados de fiebre chikungunya en México hasta la semana epidemiológica 40, 2015

Estado	Número de casos	Porcentaje (%)
Baja California Sur	45	0,52
Campeche	160	1,85
Chiapas	619	7,14
Chihuahua	1	0,01
Coahuila	7	0,08
Colima	915	10,56
Estado de México	45	0,52
Guerrero	1 593	18,38
Hidalgo	1	0,01
Jalisco	102	1,18
Michoacán	1 434	16,54
Morelos	400	4,61
Nayarit	38	0,44
Nuevo León	5	0,06
Oaxaca	1 121	12,93
Quintana Roo	72	0,83
Sinaloa	16	0,18
Sonora	6	0,07
Tabasco	30	0,35
Tamaulipas	4	0,05
Veracruz	1 139	13,14
Yucatán	915	10,56
Total	8 668	100

- Presencia del vector *Aedes aegypti* o *Aedes albopictus.*
- Antecedente de visita o residencia en áreas de transmisión en las dos semanas previas al inicio del cuadro clínico.
- Existencia de casos confirmados en la localidad.

Se consideró como caso confirmado de CHIK a todo caso sospechoso con resultado positivo a CHIKV mediante alguna de las siguientes pruebas de laboratorio específicas:

- Detección de ARN viral mediante reacción en cadena de la polimerasa con transcriptasa inversa (RT-PCR por sus siglas en inglés) en tiempo real en muestras de suero tomado en los primeros cinco días de inicio de la fiebre.
- Detección de anticuerpos en muestra de suero a partir de sexto día de iniciada la fiebre.

Se consideró como caso descartado de CHIK a todo caso en el que no se demuestre evidencia de la presencia de algún marcador serológico o virológico para CHIKV por técnicas de laboratorio reconocidas por el InDRE (6).

### Algoritmo diagnóstico

Se realizó la detección de ARN viral mediante RT-PCR durante la fase aguda de la enfermedad (0-5 días de inicio de síntomas). En fase de convalecencia (6-12 días de inicio de síntomas) se realizó la determinación de anticuerpos IgM mediante estuches comerciales y captura de anticuerpos IgM antichikungunya (MAC-ELISA).

### RESULTADOS

Se realizó la caracterización epidemiológica de fiebre chikungunya en México. El total de los casos reportados por México a la OPS al cierre del año 2014 fue de 155 casos autóctonos, 13 casos importados y ninguna defunción; se vieron afectados solo los estados de Chiapas, Guerrero, Oaxaca, Sinaloa y Sonora. Para el año 2015, el total de casos autóctonos en México para la semana epidemiológica 40 fue de 8 668 casos confirmados por laboratorio; el estado de Guerrero fue el que mayor número de casos reportó con 18,38% (1 593), seguido por Michoacán con 16,54 % (1 434) y Veracruz con 13,14%(1 139) (cuadro 1).

Durante 2015, el porcentaje de casos autóctonos de fiebre chikungunya que requirieron hospitalización debido a la gravedad de la sintomatología fue de 9% (729), de los cuales el grupo de edad más afectado fue el de 20 a 24 años. Los casos autóctonos confirmados de CHIK en México para la semana epidemiológica 40 presentaron los siguientes síntomas: fiebre en 98% de los casos (8 564), seguido por cefalea con 91,6% (7 941), mialgias 89,9% (7 792), artralgias leves 73,5% (6 367), poliartralgias graves 72,6% (6 295), exantema 58% (5 032), escalofríos56,2% (4 876), dolor retroocular 56% (4 832) y dolor de espalda 50% (4 317).

**FIGURE 1.**
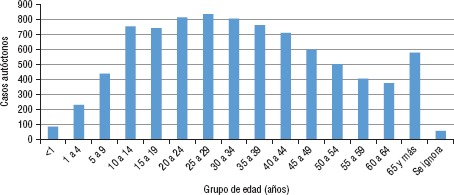
Distribución del número de casos autóctonos de fiebre chikungunya por grupo etario en diferentes estados de México hasta la semana epidemiológica 40, 2015

La distribución por sexo de los casos autóctonos confirmados de CHIK en 2015 muestra predominio del sexo femenino con 64 % (5 583). El grupo etario que ha presentado el mayor número de casos de CHIK para la semana epidemiológica 40 de 2015 es el de 25 a 29 años de edad (figura 1). Es importante mencionar que el grupo de 60 años en adelante constituye un grupo vulnerable que presentó un número significativo de casos autóctonos (figura 1).

**FIGURE 2.**
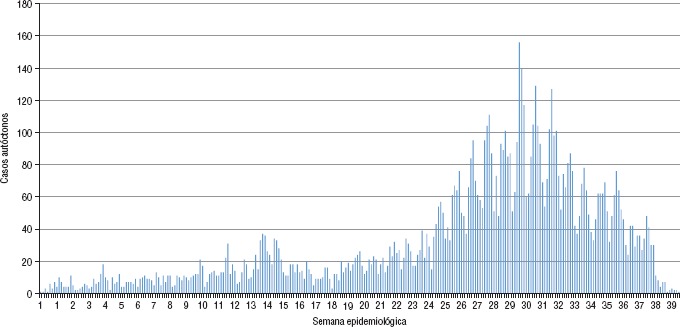
Curva epidémica de los casos autóctonos de fiebre chikungunya en México, semanas epidemiológicas 1 a 40, 2015

La curva epidémica de los casos de CHIK en México para el año 2015 mostrada en la figura 2, con corte en la semana epidemiológica 40 de acuerdo a la fecha de inicio de los síntomas, indica con claridad que los casos de fiebre chikungunya fueron en aumento con los meses, a manera de pequeños conglomerados, con un pico máximo entre julio y agosto y afectando a un total de 22 de las 32 entidades federativas del país.

## DISCUSIÓN

El 13 de junio de 2014, México notificó ante el Reglamento Sanitario Internacional (RSI) el primer caso importado de CHIKV en una mujer de 39 años, residente del municipio de Tlajomulco de Zúñiga, Jalisco, quien se dedica al deporte, con antecedente de viaje y estancia en la isla Antigua y Barbuda por un evento deportivo. La cronología de su sintomatología fue descrita por Rivera (7). El 7 de noviembre del mismo año, se reportó al RSI a través del Centro Nacional de Enlace (CNE), el primer caso confirmado autóctono en el estado de Chiapas, México; el caso corresponde a una niña de 8 años de edad, originaria de Arriaga, Chiapas, sin antecedente de viaje a otro país. Los datos sintomatológicos fueron también publicados (8).

El comportamiento de los casos de CHIK en México en cada semana epidemiológica está dentro de lo esperado, si se compara con otros brotes en América Latina, con un pico máximo en los meses de julio y agosto durante la fase epidémica.

La presentación clínica de los casos autóctonos de fiebre chikungunya en México ha mostrado algunas características diferentes a las observadas en otros brotes de los países africanos, asiáticos y otras regiones de América (9-12), con un alto porcentaje de casos con cefalea y mialgias, mayor que en otros reportes. Se esperaba que uno de los síntomas predominantes fueran las artralgias, donde en algunas publicaciones representan más de 90% en los casos, en México representa 70% e incluye formas leves, moderadas y graves.

En cuanto a la distribución por sexo, coincide con los brotes presentados en la Región, con predominio del sexo femenino. A pesar de que solo 9% de los casos requirieron hospitalización, sería importante la investigación de los días de incapacidad promedio tanto de estos casos como los que se manejan de manera ambulatoria. Esto permitiría conocer del gasto económico a nivel nacional por esta enfermedad.

Por último, a pesar de que la confirmación de casos autóctonos se realiza con base en la toma de muestra de un porcentaje de casos sospechosos, el impacto epidemiológico al conocer la caracterización de la enfermedad de esta muestra poblacional permite conocer mejor lo que está sucediendo en México para establecer políticas de salud pública que contribuyan a la prevención y el control de la enfermedad.

Es de vital importancia implementar metodologías de biología molecular para la identificación, filogenia y metagenómica de los CHIKV presentes en México. Recientemente, el InDRE publicó la secuencia genómica de dos aislamientos de CHIKV (13), por lo que se deben considerar rápidamente comparaciones filogenéticas y moleculares para identificar el origen del virus introducido, así como su caracterización molecular.

## Agradecimientos.

Los autores agradecen la información proporcionada por la Red Nacional de Laboratorios de Salud Pública y los departamentos de Epidemiología del país al Sistema Nacional de Vigilancia Epidemiológica (SiNaVE) de México.

## Declaración.

Las opiniones expresadas en este manuscrito son responsabilidad del autor y no reflejan necesariamente los criterios ni la política de la *RPSP/PAJPH* y/o de la OPS.
